# Symptomatic Premature Ventricular Contractions in Vasovagal Syncope Patients: Autonomic Modulation and Catheter Ablation

**DOI:** 10.3389/fphys.2021.653225

**Published:** 2021-05-03

**Authors:** Lihui Zheng, Wei Sun, Yu Qiao, Bingbo Hou, Jinrui Guo, Ammar Killu, Yan Yao

**Affiliations:** ^1^Fuwai Hospital, National Center for Cardiovascular Diseases, Chinese Academy of Medical Sciences and Peking Union Medical College, Beijing, China; ^2^Department of General Medicine, Monash Health, Melbourne, VIC, Australia; ^3^Department of Cardiovascular Disease, Mayo Clinic, Rochester, MN, United States

**Keywords:** vasovagal syncope, ventricular arrhythmia, autonomic modulation, deceleration capacity, catheter ablation

## Abstract

**Introduction:**

There has been limited reports about the comorbid premature ventricular contractions (PVCs) and vasovagal syncope (VVS). Deceleration capacity (DC) was demonstrated to be a quantitative evaluation to assess the cardiac vagal activity. This study sought to report the impact of autonomic modulation on symptomatic PVCs in VVS patients.

**Methods and Results:**

Twenty-six VVS patients with symptomatic idiopathic PVCs were consecutively enrolled. Identification and catheter ablation of left atrial ganglionated plexi (GP) and PVCs were performed in 26 and 20 patients, respectively. Holter 24 h-electrocardiograms were performed before and after the procedure to evaluate DC and PVCs occurrence. Eighteen patients were subtyped as DC-dependent PVCs (D-PVCs) and eight as DC-independent PVCs groups (I-PVCs). In D-PVCs group, circadian rhythm of hourly PVCs was positively correlated with hourly DC (*P* < 0.05) while there was no correlation in I-PVCs group (*P* > 0.05). Fifty-three GPs with positive vagal response were successfully elicited (2.0 ± 0.8 per patient). PVCs failed to occur spontaneously nor to be induced in six patients. In the remaining 20 patients, PVCs foci identified were all located in the ventricular outflow tract region. Post-ablation DC decreased significantly from baseline (*P* < 0.05). During mean follow-up of 10.64 ± 6.84 months, syncope recurred in one patient and PVCs recurred in another. PVCs burden of the six patients in whom neither catheter ablation nor antiarrhythmic drugs were applied demonstrated a significant decrease during follow-up (*P* = 0.037).

**Conclusion:**

Autonomic activities were involved in the occurrence of symptomatic idiopathic PVCs in some VVS patients. D-PVCs might be facilitated by increased vagal activities. Catheter ablation of GP and PVCs foci may be an effective, safe treatment in patients with concomitant VVS and idiopathic PVCs.

## Introduction

Although vasovagal syncope (VVS) and premature ventricular contractions (PVCs) are both common, there has, yet been little focus on patients with both conditions. VVS is an autonomic cardiovascular disorder featuring enhanced vagal tone and sympathetic withdrawal (Moya et al., 2009). Deceleration capacity (DC) is a quantitative evaluation index of cardiac parasympathetic tone developed by Bauer et al. (2006a,b, 2009) Our previous study demonstrated that DC provides a quantative assessment of the cardiac vagal function in VVS patients (Zheng et al., 2020), and radiofrequency (RF) catheter ablation targeting ganglionated plexi (GP) in the left atrium to treat VVS can significantly decrease the vagal tone of the cardiac autonomic function indexed by DC (Sun et al., 2016).

The impact of autonomic modulation on the genesis and maintenance of ventricular arrhythmias (VAs) remains controversial. On one hand, increased sympathetic tone has been observed preceding the occurrence of idiopathic VAs in several studies (Fei et al., 1994; Hayashi et al., 1997; Zimmermann, 2005). On the other hand, vagal activation reportedly increases the risk of idiopathic ventricular tachycardia (VT) or ventricular fibrillation (VF) in certain patients (Kasanuki et al., 1997; Mizumaki et al., 2012).

The existence of PVCs as a background to VVS provides a unique way to explore links between this cardiac autonomic disorder and PVCs. The primary goal of our study was to explore the impact of disordered autonomic regulation on the occurrence of PVCs in VVS patients via measurement of DC. Our secondary goal was to evaluate the safety and efficacy of RF catheter ablation as a therapeutic method in this patient group.

## Materials and Methods

### Study Populations

Twenty-six patients suffered from symptomatic frequent idiopathic PVCs with coexisting VVS were consecutively enrolled from January 2014 to March 2016. The clinical diagnosis of VVS was determined when syncope is triggered by emotional distress or orthostatic stress and is associated with a typical prodrome of autonomic activation (pallor, nausea, or sweating, etc.). Frequent PVCs were defined as occurring ≥30/h on Holter electrocardiogram (ECG) (Ataklte et al., 2013). Exclusion criteria were as follows:

- All other causes of syncope and transient loss of consciousness;
- Myocardial infarction within 6 months, concomitant chronic diseases including diabetes mellitus and neurological disorders;
- Previous cardiac interventions (e.g., heart surgery, catheter ablation, and permanent pacemaker implantation);
- Structural heart disease;
- Previous documented VT/VF.

All antiarrhythmic drugs were discontinued for at least 5 half-lives before the study. Tilt table test (TTT) was performed, and a positive TTT response was defined as syncope or presyncope associated with hypotension (systolic blood pressure <70 mmHg or diastolic blood pressure <40 mmHg) or bradycardia (heart rate <40 bpm). Subjects with positive TTT response were classified as mixed, cardio-inhibitory or vasodepressor subtype (Brignole et al., 2000).

The Local Ethics Committee approved the study and written informed consent was obtained from each participant at recruitment.

### Holter Monitoring

Twelve-channel 24-h Holter ECG data was obtained using MIC-12H Holter Monitoring System (Jinke Instruments, Beijing, China) at baseline and 1 month after the PVCs and VVS ablation procedure. Patients maintained normal daily activities other than strenuous exercise during the recording period. After uploading the ECG data to MIC-12H Analysis Platform, QRS classification (normal, PVCs, supraventricular premature beat) was automatically computed, manually reviewed, and corrected when necessary. VAs analysis, DC measurement and heart rate variability (HRV) analysis were then conducted.

### Deceleration Capacity Analysis

Deceleration capacity was calculated automatically using the phased-rectified signal averaging (PRSA) technique (Bauer et al., 2006b). The protocol was divided into 4 steps.

**Step 1:** All sinus R–R intervals obtained from the 24-h Holter recording data were aligned. R–R intervals longer than the preceding R–R were recognized as “anchors”. R–R intervals of >5% longer duration were excluded to avoid artifact errors.

**Step 2:** Segments containing 15 R–R intervals preceding the anchor R–R and 15 R–R intervals following the anchor R–R were defined.

**Step 3:** All the defined segments were aligned and overlapped.

**Step 4:** Each signal within the aligned segments was averaged and DC was calculated using the equation:

$$\text{DC}\,\left[X_{0}+X_{1}-X_{-1}-X_{-2}\right]/4,$$

where *X*_0_ and *X*_1_ represent the averages of the anchors and of the following R–R intervals. *X*_–1_ and *X*_–2_ represent the two R–R intervals preceding the anchors.

Overall DC (ODC) for 24 h, daytime DC (DDC) from 06:00 to 23:00, and nighttime DC (NDC) from 23:00 to 06:00 were calculated, respectively. Further, hourly DC values were automatically generated for each hour during a 24-h period. DC measurements was applicated in our previous study to quantitatively assess the cardiac parasympathetic tone in patients with VVS (Sun et al., 2016; Zheng et al., 2020).

Basing on the association between hourly PVCs burden and hourly DC, the PVCs can be defined as DC-dependent PVCs (D-PVC) when there is a statistically significant association between hourly PVCs and hourly DC. Otherwise, PVCs would be defined as DC-independent PVCs (I-PVC) when there is no statistically significant relationship between the two indexes.

### PVCs and Heart Rate Variability Analysis

Numbers of PVCs from Holter ECG data were calculated with MIC-12H Analysis Platform. PVCs burden was defined as PVCs percentage of total beats in a given period.

Baseline spectral HRV of 5-min segments free of PVCs, including the following components of high frequency (HF, 0.15–0.4 Hz), low frequency (LF, 0.04–0.15 Hz) and LF to HF ration (LF/HF) were computed through the MIC-12H Analysis Software. HF was applied as an index of parasympathetic activity while LF and LF/HF were used as the indices of sympathetic activity (Sassi et al., 2015).

### Mapping and Ablation for VVS and PVCs

If possible, both the PVCs and VVS were targeted for ablation during the same procedure.

#### Endocardial Left Atrial Ganglionated Plexi Ablation for VVS

An anatomical-guided GP ablation approach was performed in all 26 subjects in accordance with our previous report in detail (Sun et al., 2016). Five GP sites in the left atrium were attempted in each procedure, including left superior GP (LSGP), left inferior GP (LIGP), right anterior GP (RAGP), right inferior GP (RIGP), and left lateral GP (LLGP). A 4mm catheter (Safire, Abbott, TX, United States) was used to deliver RF energy at targeted sites. The temperature and power limits were 60°C and 60 W, respectively. A positive vagal response (VR) was defined when RF energy induced any of the following phenomena within 10 s: transient ventricular asystole, atrioventricular block, or an increase in mean R–R interval of 50%. The endpoint of the anatomical-guided procedure was defined as once at each GP site five consecutive ablation attempts failed to induce any VR (Sun et al., 2016).

A 4 mm catheter (Safire, Abbott, TX, United States) was applied for RF energy delivery in a temperature-controlled mode. The maximal delivered temperature was 60°C and maximal power was 40 W.

### Mapping and RF Catheter Ablation for PVCs

premature ventricular contractions did not occur spontaneously in six patients. Further intravenous isoprenaline failed to induce any PVCs in these six subjects. Therefore, PVCs mapping and ablation were performed only for the other 20 patients during the same procedure of GP ablation. The identification of arrhythmogenic foci was achieved using a three-dimensional mapping system (Ensite NavX, Abbott, TX, United States). The target lesion was recognized at the site where the local activation signal was the earliest and/or at least 11 of 12 leads QRS morphologies of paced PVCs matched the clinical PVCs.

A 4 mm catheter (Safire, Abbott, TX, United States) was applied for RF energy delivery in a temperature-controlled mode. The maximal delivered temperature was 60°C and maximal power was 40 W. If PVCs were inhibited within 30 s, five additional lesions around the target site were created. Acute success was defined when spontaneous or inducible PVCs did not recur for 30 min after ablation.

### Follow-Up

No antiarrhythmic drugs for PVCs were routinely administered after the procedure. Pharmacological and non-pharmacological treatment for VVS were withheld after the catheter ablation. Patients were followed up in our outpatient clinic at 1 month, which included 24-h Holter yielding DC and PVCs occurrence post-ablation. Subsequently, patients were contacted by telephone for recurrent PVCs when they were unable to attend the hospital. PVCs recurrence was defined when PVCs were >1000 and confirmed by morphology criteria on a 24-h Holter (Flemming et al., 1999). Recurrent VVS was carefully documented. Prodromes including transient dizziness, diaphoresis or fatigue without loss of consciousness were not considered as recurrent syncope.

### Statistical Analysis

Data with a normal distribution are expressed as mean ± SD. The baseline associations between hourly PVCs burden and hourly DC, LF, HF, LF/HF, and mean heart rate (HR) were analyzed using Pearson’s correlation. PVCs burden and DC changes after catheter ablation were performed using paired Student’s *t*-test. Statistical significance was considered as *P* < 0.05. Statistical analyses were carried out using SPSS software (version 19.0, SPSS Inc., Chicago, IL, United States).

## Results

### Baseline Clinical Characteristics of the Study Population

According to the association between hourly PVCs burden and hourly DC, the enrolled patients were classified into D-PVCs (*n* = 18) and I-PVCs groups (*n* = 8). In this study we found no case where hourly PVCs were negatively associated with hourly DC. Their detailed general characteristics were shown in Table 1. Average number of syncopal episodes was 4.4 ± 3.0 during the 6 months before enrollment. All subjects demonstrated a positive response to TTT. There were no significant differences observed in age, gender, body mass index, blood pressure, syncopal episodes, TTT results, and echocardiographic parameters between the two groups. Patients’ cardiac vagal tone was reflected by ODC, DDC, and NDC, respectively.

**TABLE 1:** General clinical characteristics of the enrolled patients.

	**Total cohort (*N* = 26)**	**D-PVCs (*n* = 18)**	**I-PVCs (*n* = 8)**	***P*-value**
**General parameters**				
Age (years)	41.8 ± 15.4	42.1 ± 17.3	39.6 ± 12.5	0.614
Female (*n*)	14/26	10/18	4/8	0.793
BMI (kg/m^2^)	22.7 ± 3.7	23.1 ± 3.3	21.8 ± 5.2	0.674
Systolic BP (mmHg)	118 ± 16	121 ± 12	119 ± 15	0.374
Diastolic BP (mmHg)	75 ± 8	72 ± 6	78 ± 11	0.487
**VVS associated parameters**				
Syncopal episodes*	4.4 ± 3.0	4.3 ± 2.7	4.6 ± 3.4	0.773
Supine heart rate	71.4 ± 11.7	70.9 ± 13.2	72.1 ± 10.5	0.523
Patients who reported prodromes, *n*	21/26	15/18	6/8	0.619
**Identifiable triggers**				
Long-term standing	20/26	14/18	6/8	0.877
Pain	5/26	3/18	2/8	0.619
Fear	1/26	1/18	0/8	0.497
TTT positive	26/26	18/18	8/8	–
Mixed	13/26	8/18	5/8	0.395
Cardio-inhibitory	6/26	4/18	2/8	0.877
Vaso depressive	7/26	5/18	2/8	0.883
**Echocardio graphic parameter**				
LAD (mm)	31.1 ± 3.6	30.8 ± 2.7	31.4 ± 3.4	0.613
LVEDD (mm)	43.4 ± 2.8	42.7 ± 3.1	43.7 ± 3.4	0.712
LVEF (%)	64.8 ± 4.3	65.3 ± 5.1	64.5 ± 3.9	0.682
**Deceleration capacity measurement**				
ODC (ms)	10.39 ± 3.23	10.76 ± 3.27	9.71 ± 2.93	0.367
DDC (ms)	9.43 ± 2.79	9.62 ± 3.16	9.35 ± 2.76	0.716
NDC (ms)	12.96 ± 4.15	13.59 ± 4.67	9.94 ± 3.96	0.087

*Syncopal episodes were counted during the six months prior to enrollment.

*VVS, vasovagal syncope; TTT, tilt table testing; LAD, left atrium diameter; LVEDD, left ventricle end diastolic diameter; LVEF, left ventricular ejection fraction; ODC, overall deceleration capacity; DDC, daytime deceleration capacity; NDC, nighttime deceleration capacity; BMI, body mass index; BP, blood pressure; PVCs, premature ventricular contractions. D-PVCs were when hourly PVCs were positively correlated with hourly DC (*P* < 0.05). I-PVCs were those that had no statistical relationship with hourly DC.*

### Baseline Vagal Tone and PVCs Occurrence

**FIGURE 1:**
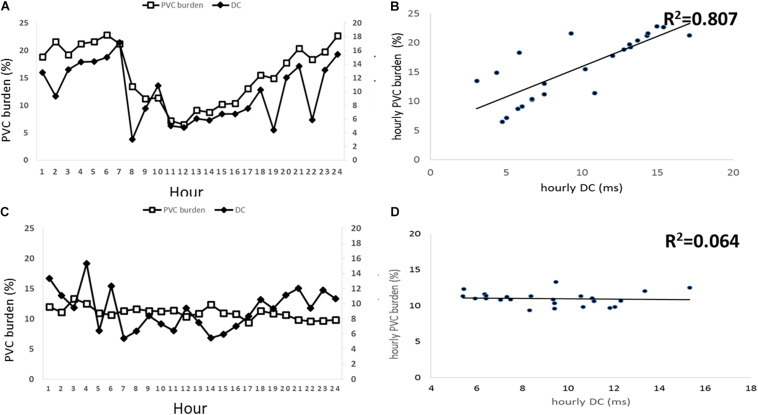
Associations between hourly premature ventricular contractions (PVCs) burden in % and hourly deceleration capacity (DC) in ms. The left two panels **(A,B)** illustrate this association in patients with PVCs dependent on DC and the right two panels illustrate the association in DC independent PVCs patients. **(A)** shows hourly PVCs burden and hourly DC plotted over 24 h in D-PVCs patients. **(B)** shows correlation between hourly PVCs burden and hourly DC in D-PVC patients. **(C)** shows hourly PVCs burden and hourly DC plotted over 24 h in I-PVC patients. **(D)** shows correlation between hourly PVCs burden and hourly DC in I-PVC patients.

Although DC parameters were identical between D-PVCs and I-PVCs patients, the cardiac autonomic nervous system was shown to have different impacts on the occurrence of PVCs. As illustrated in Figure 1, similar patterns of circadian rhythm of hourly PVCs burden and hourly DC were observed in the D-PVCs group. On the other hand, hourly variation in PVCs burden was not reflected by hourly DC in the I-PVC s group. To investigate further the association between hourly PVCs burden and cardiac autonomic tone, hourly HRV indices and hourly HR were analyzed. In the D-PVCs group, hourly PVCs burden was negatively correlated with hourly HR in each patient (*P* < 0.05). HF was positively correlated with hourly PVCs burden (*P* < 0.05) while LF and LF/HF were negatively associated with PVCs burden in some patients (*P* < 0.05). However, in the I-PVCs group, no statistical correlation could be found between hourly PVCs burden and hourly DC, neither in hourly HR and HRV indices (*P* > 0.05) (Table 2).

**TABLE 2:** Correlations between hourly PVCs burden and hourly DC, HR, and HRV in each patient.

**Groups**	**Patient number**	**Correlation coefficient**				
		**DC**	**HR**	**LF**	**HF**	**LF/HF**
D-PVCs	1	0.807**	−0.789**	0.240	0.569*	−0.583*
	2	0.796**	−0.673*	0.204	0.496*	−0.14
	3	0.879**	−0.734**	0.228	0.679*	−0.337
	4	0.898**	−0.820**	−0.573*	0.214	−0.210
	5	0.629*	−0.757**	0.211	0.213	−0.406*
	6	0.572*	−0.814**	0.228	0.534*	−0.481*
	7	0.719**	−0.669*	−0.339	0.617*	−0.575*
	8	0.799**	−0.838**	0.207	0.250	−0.412*
	9	0.613*	−0.574*	0.519*	0.307	−0.601*
	10	0.771**	−0.696*	0.209	0.613*	0.122
	11	0.879**	−0.873**	0.216	0.787*	−0.493*
	12	0.684*	−0.762**	−0.271	0.475	−0.506*
	13	0.691*	−0.572*	0.015	0.037	−0.093
	14	0.769**	−0.806**	−0.470*	0.527*	−0.674*
	15	0.843**	−0.715**	0.196	0.328	−0.247
	16	0.652*	−0.518*	0.287	0.429	−0.104
	17	0.621*	−0.570*	0.306	0.445	−0.138
	18	0.722**	−0.625*	0.054	0.616*	0.107
I-PVCs	1	−0.214	0.196	0.132	0.207	0.103
	2	0.037	−0.013	0.104	0.096	0.114
	3	−0.071	0.061	0.097	0.132	0.148
	4	0.064	0.039	0.307	0.162	0.005
	5	0.010	−0.017	−0.046	0.019	0.177
	6	−0.109	0.243	0.147	0.034	−0.039
	7	0.116	−0.079	0.209	0.193	−0.128
	8	−0.147	0.183	0.154	0.085	0206

*P < 0.05, **P < 0.01.

*PVCs, premature ventricular contractions; DC, deceleration capacity; HR, heart rhythm; HRV, heart rate variability; LF, low frequency; HF, high frequency; D-PVCs and I-PVCs as in Table 1.*

### Catheter Ablation of GP and PVCs

A positive VR was elicited in 40.7% (53/130) of the GP sites. The LSGP was the most common GP site where a positive VR was observed (24/26, 92.3%), followed by RAGP (11/26, 42.3%), LIGP (8/26, 30.7%), LLGP (5/26, 19.2%), and RIGP (5/26, 19.2%). The average number of positive VR GP sites was 2.0 ± 0.8 per patient.

Among the 20 patients who underwent PVCs mapping and ablation, 12 patients were from the D-PVCs group while eight were from the I-PVCs group. The coupling interval and QRS duration of PVCs were identical between the two groups (*P* = 0.826). PVCs foci were determined concurrently using both activation mapping and pace-mapping in all 20 patients. All immediately successful targets were located in the ventricular outflow tracts, with right ventricular outflow tract (RVOT) septum in 19 patients and left aortic sinus of Valsalva in 1 (Table 3). Acute success was achieved in all patients. No procedure related complications, including vascular complications, pericarditis, and tamponade occurred.

**TABLE 3:** Electrophysiological features of PVCs in D-PVC and I-PVC patients.

	D-PVCs (*n* = 12)	I-PVCs (*n* = 8)	*P*-value
**Arrhythmogenic foci**			
RVOT septum	11/12	8/8	0.402
Left coronary sinus	1/12	0/8	0.402
Coupling interval (ms)	472.6 ± 22.8	466.9 ± 19.9	0.502
PVCs QRS duration (ms)	146.5 ± 8.3	144.9 ± 6.7	0.692
**Mapping system used**			
Ensite NavX	12/12	8/8	–
**Mapping strategies**			
Activation mapping + pace-mapping	12/12	8/8	–
Earliest activation time preceding PVCs (ms)	38.5 ± 7.2	39.6 ± 6.9	0.826
Qualified pace-mapping	10/12	6/8	0.648

*PVCs, premature ventricular contractions; RVOT, right ventricular outflow tract; D-PVCs and I-PVCs as in Table 1.*

### Post Procedure Changes in PVCs Burden and DC

Detailed information was shown in Table 4. In the 20 patients who underwent both the left atrial GP ablation and PVCs ablation, post-procedural ODC, DDC, and NDC demonstrated significant reduction compared with baseline (*P* < 0.001). PVCs burden decreased from 247.26 ± 97.84 to 9.14 ± 26.62‰ (*P* < 0.001). In the six patients who underwent only the left atrial GP ablation, ODC, DDC, and NDC also differed significantly from the baseline (*P* = 0.017, *P* = 0.026, and *P* < 0.001), respectively. Besides, although PVCs still exist, a reduction of PVCs burden in this patient group was observed from 266.44 ± 127.60 to 89.35 ± 61.28‰ (*P* = 0.037). During a mean follow-up period of 10.64 ± 6.84 (1–26) months, one patient from I-PVCs group who underwent catheter ablation for both PVCs and VVS experienced recurrent PVCs, while VVS recurred in another patient from D-PVCs group who underwent both procedures.

**TABLE 4:** Effect of catheter ablation on PVCs burden and DC.

	**GP ablation + PVC ablation (*n* = 20)**			**GP ablation alone (*n* = 6)**		
	**Baseline**	**1 month Post-ablation**	***P*-value**	**Baseline**	**1 month Post-ablation**	***P*-value**
ODC (ms)	10.94 ± 3.35	3.26 ± 2.72	<0.001	10.54 ± 4.02	4.32 ± 3.63	0.017
DDC (ms)	9.76 ± 4.01	2.98 ± 3.01	<0.001	9.33 ± 2.87	3.55 ± 2.62	0.026
NDC (ms)	12.72 ± 4.54	4.33 ± 3.64	<0.001	13.89 ± 2.64	4.20 ± 3.23	<0.001
PVC burden (‰)	247.26 ± 97.84	9.14 ± 26.52	<0.001	266.44 ± 127.60	89.35 ± 61.28	0.037
PVC number	23,475.4 ± 10,152.2	456.5 ± 1030.3	<0.001	21,927.5 ± 10,132.2	6278.0 ± 3892.7	0.004

*PVCs, premature ventricular contractions; DC, deceleration capacity; GP, ganglionated plexi; D-PVC & I-PVC as in Table 1; ODC, overall deceleration capacity; DDC, daytime deceleration capacity; NDC, nighttime deceleration capacity.*

## Discussion

### Major Findings

To the best of our knowledge, there is limited study to address symptomatic idiopathic PVCs in VVS subjects. Hourly PVCs burden in VVS patients demonstrated two different circadian rhythm patterns. In the vagal-dependent group, hourly PVCs burden and hourly DC showed similar circadian fluctuation patterns, which were positively associated. In contrast, in the vagal-independent group, PVCs were independent of DC. Interestingly, PVCs burden decreased in patients who underwent only GP ablation. In other words, vagal activity facilitated the occurrence of PVCs and left atrial autonomic modification reduced PVCs burden in some VVS patients.

Secondly, endocardial left atrial GP ablation and PVCs ablation could be successfully performed during the same procedure for VVS patients with concomitant symptomatic PVCs.

### Autonomic Modulation of Ventricular Arrhythmias in General and in VVS Patients

Recent studies have indicated that autonomic tone could influence both the genesis and maintenance of VAs. In most cases, VAs are activated or aggravated by sympathetic dominance (Vanoli et al., 1991; Fei et al., 1994; Hayashi et al., 1997; Inagaki et al., 2005; Zimmermann, 2005). According to Hasdemir et al. (2009), HF stimulation (200 Hz/0.3 ms pulse duration) in the left pulmonary artery during dobutamine infusion successfully induced VAs exhibiting Left bundle branch block-morphology and inferior axis in 66.7% of healthy volunteers. Treatments that improve vagal tone or decrease sympathetic tone have been shown to have protective effect against ischemia induced VT/VF in animal models (Vanoli et al., 1991; Lujan et al., 2010). However, other studies presented different views inferring that VAs could be facilitated by vagal activation both in animals and in humans (Kasanuki et al., 1997; Farkas et al., 2008; Mizumaki et al., 2012). Typically in idiopathic VF patients, the increased vagal activity has been correlated with strong augmentation of J-wave elevation during bradycardia, which was used to explain the predominant occurrence of VF at night (Mizumaki et al., 2012).

This study was performed on a special group of patients suffering from VVS, a cardiac autonomic regulation disorder with greatly increased vagal tone as its outstanding characteristic (Zheng et al., 2020). Using PRSA technique, DC measurements reflect the general cyclical changes in sinus heart rhythm without the influence from physiological process like respiratory or baroreflex. Therefore, DC provides quantitative assessment of the cardiac vagal tone. In our previous study, DC was applied to quantitatively evaluate the abnormal vagal function in patients with VVS, which demonstrated an increased vagal tone even during asymptomatic period compared with normal controls (Zheng et al., 2020). Besides, most patients did not experience recurrent syncope after catheter ablation of left atrium GPs and their DC values significantly decreased after the procedure (Sun et al., 2016). These studies implied that DC could be used as a reliable index representing vagal tone for VVS patients in this study.

In this study, PVCs occurrence exhibited different responses to vagal activity regarding the correlation between hourly PVCs burden and hourly DC. In D-PVCs patients, PVCs occurred more frequently when DC increased and less frequently when DC decreased, indicating that vagal activity promoted the genesis and maintenance of VAs. The possible mechanism of vagal modulation on VAs could be explained by triggered activity induced by early after-depolarization (EAD) (Weiss et al., 2010). Heart rate became slower when vagal activity increased; slower HR facilitates occurrence of EADs and may result in genesis of EAD associated PVCs (Zhang et al., 2020). Besides, in six patients who only received the GP ablation and discontinued antiarrhythmic drugs after the procedure, PVCs burden decreased in concomitant with the elevation of DC parameters. This phenomenon further supports our hypothesis that vagal activity influences the VAs in some VVS patients.

### Electrophysiological Findings of Symptomatic PVCs in VVS Patients

This pilot study concerning symptomatic PVCs in VVS patients presents some interesting features. First, arrhythmogenic PVCs foci were all located in the outflow tract, most of which were in the septal side of RVOT. Second, acute success of PVCs was achieved in all patients with recurrence in one patient comparable with other studies reporting PVCs ablation (0–18%) (Ribbing et al., 2003; Joshi and Wilber, 2005; Chung et al., 2014).

### Study Limitations

This study is a single center design and has a small sample size. Thus, it must be regarded as a pilot study. Besides, there was no patients in which hourly PVCs were negatively associated with hourly DC in the current study. Therefore, the current classification of D-PVCs and I-PVCs should be considered tentative as it is possible that in future studies different relationship between PVCs and DC may occur. Besides, the conclusion of autonomic modulation on PVCs occurrence should be taken with precaution and not be generalized because there were still a group of patients in whom no statistical relationship was established between DC and PVC burden, as well as the small sample size. These further studies must also attempt to understand more fully the electrophysiological mechanisms linking autonomic tone and occurrence of PVCs.

## Conclusion

Symptomatic idiopathic PVCs may be modulated by vagal activity based on their dependency on vagal tone which indexed by DC. Up to 70% of symptomatic PVCs in VVS patients could be facilitated by parasympathetic activation. In this patient population combined with PVCs and VVS, RVOT is the most common arrhythmogenic focus location and catheter ablation appears to be a safe and effective therapeutic choice.

## Data Availability Statement

The original contributions presented in the study are included in the article/Supplementary Material, further inquiries can be directed to the corresponding author/s.

## Ethics Statement

The studies involving human participants were reviewed and approved by the Ethics Committee of Fuwai Hospital, CAMS & PUMC. The patients/participants provided their written informed consent to participate in this study.

## Author Contributions

LZ and WS contributed to the study design, data collection, analysis, and writing of the manuscript. YQ, BH, and JG contributed to patient enrollment and follow-up. AK contributed to the revising of the manuscript. YY contributed to the study conception, catheter ablation, and revising of the manuscript. All authors contributed to the article and approved the submitted version.

## Conflict of Interest

The authors declare that the research was conducted in the absence of any commercial or financial relationships that could be construed as a potential conflict of interest.

## Supplementary Material

Supplementary Figure 1 Study population and subgroups illustration.

Supplementary Figure 2 Comparison of PVC numbers before and after procedure of the six patient who underwent only VVS ablation.
