# Supplementary figures and images for: Symptomatic Premature Ventricular Contractions in Vasovagal Syncope Patients: Autonomic Modulation and Catheter Ablation
Source: Front Physiol. 2021 May 3;12:653225. doi: 10.3389/fphys.2021.653225 (PMC8126685; doi:10.3389/fphys.2021.653225)

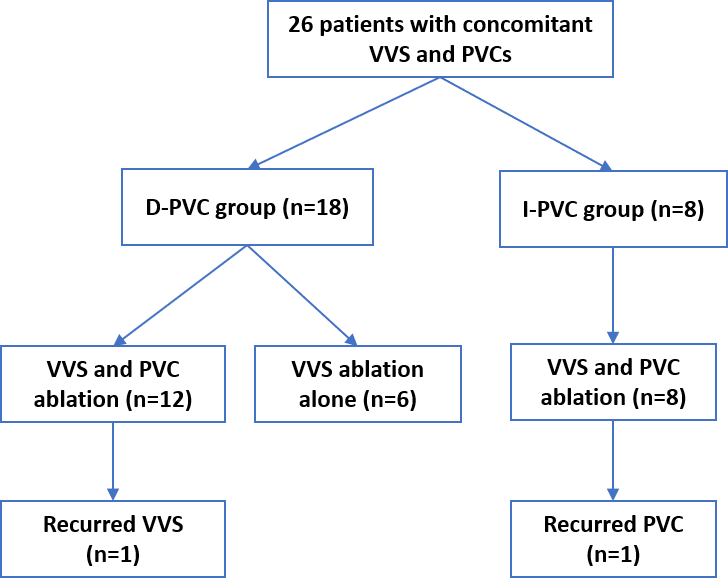

Supplement: Supplementary Figure 1 — Study population and subgroups illustration. [file Image_1.tif]

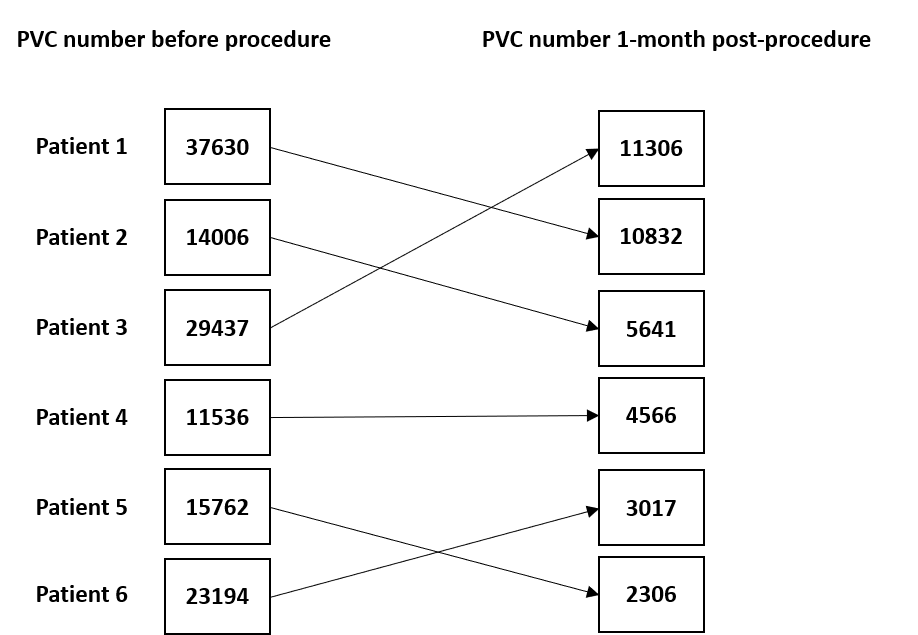

Supplement: Supplementary Figure 2 — Comparison of PVC numbers before and after procedure of the six patient who underwent only VVS ablation. [file Image_2.tif]
